# The Impact of Phase-Specific Macrophage Depletion on Intestinal Anastomotic Healing

**DOI:** 10.3390/cells12071039

**Published:** 2023-03-29

**Authors:** Maximiliane Winter, Barbara Heitplatz, Nils Koppers, Annika Mohr, Alexander D. Bungert, Mazen A. Juratli, Benjamin Strücker, Georg Varga, Andreas Pascher, Felix Becker

**Affiliations:** 1 Department of General, Visceral and Transplant Surgery, University Hospital Münster, 48149 Münster, Germany; 2 Gerhard Domagk Institute of Pathology, University Hospital Münster, 48149 Münster, Germany; 3 Core Facility Genomik, Medical Faculty Münster, Westfälische Wilhelms-University, 48149 Münster, Germany; 4 Department of Pediatric Rheumatology and Immunology, University Hospital Münster, 48149 Münster, Germany

**Keywords:** anastomotic healing, DTR, IBD, inflammation, intestine, macrophages, monocytes, mucosal inflammation, wound healing

## Abstract

Intestinal anastomotic healing (AH) is critical in colorectal surgery, since disruptive AH leads to anastomotic leakage, a feared postoperative complication. Macrophages are innate immune cells and are instrumental in orchestrating intestinal wound healing, displaying a functional dichotomy as effectors of both tissue injury and repair. The aim of this study was to investigate the phase-specific function and plasticity of macrophages during intestinal AH. Transgenic CD11b diphtheria toxin receptor (CD11b-DTR) mice were used to deplete intestinal macrophages in a temporally controlled manner. Distal colonic end-to-end anastomoses were created in CD11b-DTR, and wild-type mice and macrophages were selectively depleted during either the inflammatory (day 0–3), proliferative (day 4–10), or reparative (day 11–20) phase of intestinal AH, respectively. For each time point, histological and functional analysis as well as gene set enrichment analysis (GSEA) of RNA-sequencing data were performed. Macrophage depletion during the inflammatory phase significantly reduced the associated inflammatory state without compromising microscopic AH. When intestinal macrophages were depleted during the proliferative phase, AH was improved, despite significantly reduced perianastomotic neoangiogenesis. Lastly, macrophages were depleted during the reparative phase and GSEA revealed macrophage-dependent pathways involved in collagen remodeling, cell proliferation, and extracellular matrix composition. However, AH remained comparable at this late timepoint. These results demonstrate that during intestinal AH, macrophages elicit phase-specific effects, and that therapeutic interventions must critically balance their dual and timely defined role.

## 1. Introduction

Intestinal wound healing is a tightly regulated and complex process, encompassing superficial re-epithelialization (epithelial restitution), mucosal wound healing of deeper defects (e.g., ulcers formed in inflammatory bowel disease (IBD)) as well as anastomotic healing (AH) following intestinal resection. Disruptive AH consequently leads to anastomotic leakage (AL), a common but feared postoperative complication, associated with significant morbidity and mortality. However, despite the high prevalence (1–19%) of AL, the current understanding of intestinal wound healing remains remarkably limited [1]. Regarding the clinical impact of AL, there is an urgent unmet need to further unravel fundamental and mechanistic pathways in AH to understand, prevent and treat impaired intestinal wound healing [2].

In humans and mice, AH proceeds via an overlapping pattern of events, classically divided into three merging stages: (I) an inflammatory, (II) a proliferative, and (III) a reparative phase [3]. Each phase is temporally defined and characterized by specific hallmark events, elicited by dynamic interactions between resident immune, endothelial, stroma, and epithelial cells (EC), as well as infiltrated leukocytes [4]. Among them, macrophages (MΦ) represent a key subset. The intestinal mucosa represents human’s largest reservoir for MΦ [5], which are particularly abundant in the lamina propria (LP) of the colon. Most intestinal MΦ are constantly replenished by monocytes, which enter the gut and differentiate locally into mature, anti-inflammatory MΦ [6]. However, MΦ precursors display immense plasticity and can give rise to MΦ with various phenotypes depending on tissue-specific signals [7]. They can differentiate into a spectrum of classically activated pro-inflammatory (M1-like) or alternatively activated, tissue-protective, and pro-resolving (M2-like) MΦ. This niche model of monocyte-to-intestinal-MΦ differentiation demonstrates that the fate and function of MΦ depend on their micro-environment [8], especially during inflammation and tissue trauma. Since each phase of intestinal AH is characterized by a set of specific events, and consequently, vast changes in the intestinal micro-environment, MΦ play a crucial yet dichotomous role, as they display an immense phase-specific function and plasticity [9].

The origin of intestinal wound healing is a robust inflammatory response. Tissue-resident and especially migrated monocyte-derived MΦ elicit an inflammatory state to destroy and remove pathogens, necrotic tissue as well as debris [10,11]. Having acquired an inflammatory (M1-like) phenotype, MΦ produce reactive oxygen species (ROS), interleukins (IL-1β-6, -8, -12, and -23), and tumor necrosis factor (TNF) to induce inflammation and antimicrobial immunity. In addition, MΦ secrete proteases (e.g., matrix metalloproteinases (MMPs-2 and MMPs-9) to degrade the extracellular matrix (ECM), thereby facilitating the recruitment of inflammatory cells to the site of tissue injury, attracted by cytokines and growth factors [12].

Beginning in the proliferative phase, MΦ undergo a phenotype switch to acquire a pro-resolving and wound-healing phenotype [13,14]. Upon efferocytosis of apoptotic neutrophils and in response to IL-4 or -13 signaling [15], MΦ switch their phenotype from pro-inflammatory (M1-like) to pro-resolving (M2-like) [15]. In addition, apoptosis [16], phagocytosis [17] and the changes in the microenvironment that favor the differentiation of infiltrating monocytes into resident MΦ with non-inflammatory gene expression profiles further transform the intestinal MΦ pool during the proliferative phase of intestinal AH [18,19]. Another critical MΦ-elicited mechanism is the induction of angiogenesis via the secretion of vascular endothelial growth factor A (VEGF-A) to establish a microvascular network throughout the granulation tissue and to accelerate wound healing as well as enhance anastomotic strength [20,21].

During the reparative phase, MΦ comprise the dominant cell line for collagen synthesis and ECM remodeling, elicited by soluble growth factors [4] including transforming growth factor beta 1 (*TGF-β1*), platelet-derived growth factor (PDGF), and epidermal growth factor (EGF), which also induce intestinal epithelial cell proliferation and myofibroblast activation [22]. The composition and arrangement of collagen are further adjusted to reach typical tearing strength accompanied by the transition from provisional wound closure to a colonic wall continuum [3].

**Figure 1:**
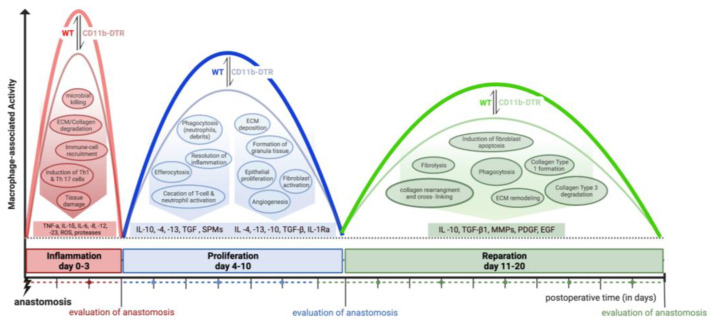
Graphical overview of the study design and macrophage-associated activities during intestinal anastomotic healing. Wild type (WT), CD11b diphtheria toxin receptor (CD11b-DTR), extracellular matrix (ECM), tumor necrosis factor alpha (TNF-α), interleukin (IL1-ß, -4, -6, -8, -10, -12, -13, -23), reactive oxygen species (ROS), transforming growth factor (TGF), specialized pro-resolving mediator (SPM), matrix metalloproteinases (MMP), platelet-derived growth factor (PDGF), epidermal growth factor (EGF).

Considering the dramatic changes in local intestinal signaling pathways associated with AH, it is reasonable to suggest a distinct plasticity in MΦ function during the three phases of intestinal AH. Thus, this study aims to define phase-specific effects of MΦ in intestinal AH by using an animal model of colorectal anastomosis in combination with innovative on-demand MΦ ablation, which allows us to specifically study phase- and cell-specific pathways (Figure 1). It was hypothesized that MΦ possess diverse roles during different phases of intestinal AH, and that MΦ depletion elicits distinct and phase-specific effects on inflammation, proliferation, and regeneration during intestinal AH (Figure 1).

## 2. Materials and Methods

### 2.1. Mice

All procedures were approved by the State Office for Nature, Environment and Consumer Protection of North Rhine-Westphalia (reference number 81-02.04. 2020.A227) and conducted in accordance with German Animal Welfare Law. All experiments were performed using male (20 to 30 g) wild-type (WT) C57BL/6 (Charles River Laboratories, Sulzfeld, Germany) and transgenic CD11b-DTR (B6.FVB-Tg (ITGAM-DTR/EGFP)34 Lan/J, Jackson Laboratory, stock #006000, Bar Harbor, ME, USA, [23]) mice. Mice were maintained at the local animal facility for at least one week for acclimatization before undergoing treatment. Animals had ad *libitum* access to food and water and were kept under pathogen-free conditions and at a constant temperature (22 ± 2 °C) and air humidity (45–65%), with a 12 h light/dark cycle.

### 2.2. CD11b-DTR Mouse Model and Monocyte and MΦ Ablation

The CD11b-DTR mouse carries a transgene insert, which codes for a simian diphtheria toxin receptor (DTR) regulated by the human integrin alpha M (ITGAM) promoter (CD11b). While WT mice are naturally resistant to diphtheria toxin (DT), the “knock-in” of DTR allows for the specific systemic and local ablation of monocytes and MΦ by injecting DT from *Corynebacterium diphtheriae*. Other immune cells, e.g., neutrophils, eosinophils, and lymphocytes, remain unaffected by DT [23]. All CD11b-DTR mice received DT (25 ng/g body weight; intraperitoneally (i.p.)), Sigma-Aldrich, St. Louis, MO, USA) suspended in phosphate-buffered saline (PBS, Sigma-Aldrich), while WT mice were injected i.p. with vehicle control (PBS, 0.01 mL/g body weight).

### 2.3. Experimental Design

**Figure 2:**
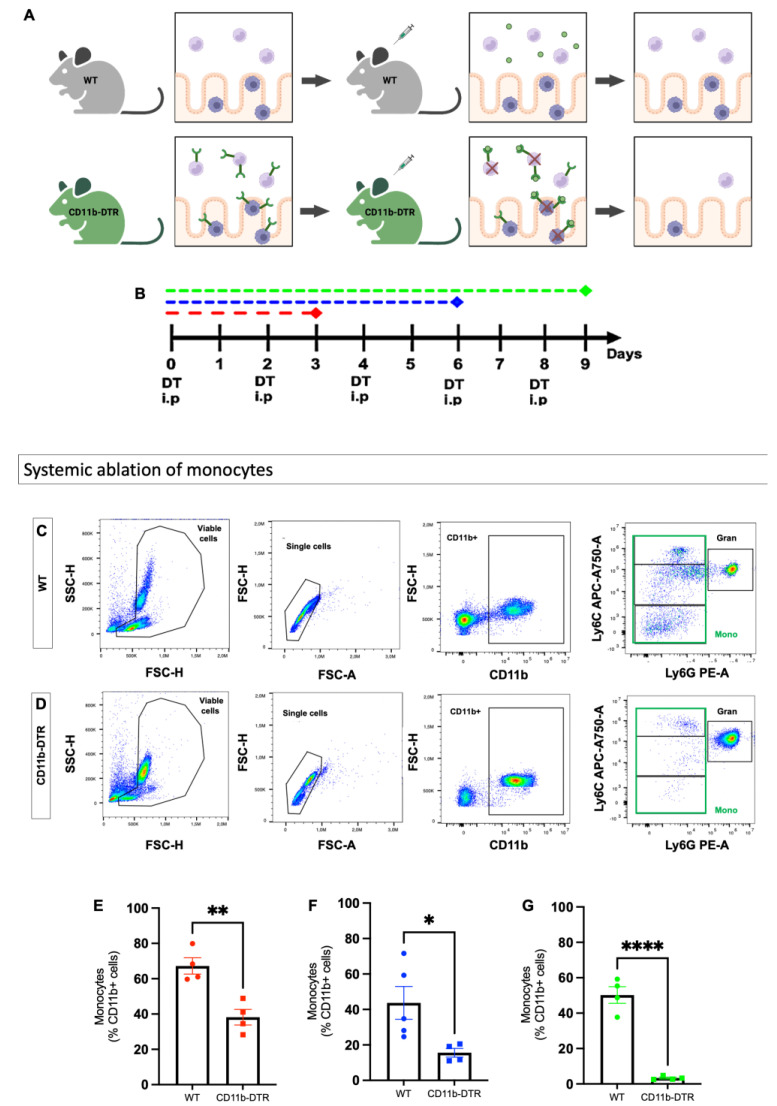
Temporally controlled diphtheria-toxin-induced monocyte depletion. (**A**) Diphtheria toxin receptor (DTR) in CD11b-DTR mice allows the specific systemic and local ablation of monocytes and macrophages by injecting diphtheria toxin (DT) intraperitoneally (i.p.), while wildtype (WT) mice are naturally resistant. (**B**) DT treatment scheme with a varying duration of DT treatment. Mice were treated with DT for 3 (red), 6 (blue) or 9 (green) days. Gating strategy for identification of circulating monocytes (mono) in (**C**) WT and (**D**) CD11b-DTR mice; representative flow cytometry plots are shown. Viable cells are identified on a forward scatter-height (FSC-H) x side scatter (SSC-H) plot, while single cells are identified on an FSC-H x FSC-area (**A**) plot. Next, CD11b x FCH-H gating was used to identify CD11+ positive cells, which were gated in two parameter density plots with Ly6G and Ly6C. Monocytes were defined as CD11b^+^Ly6G^low^Ly6C^+^cells. Numeric data for circulating monocytes subsets mice following (**E**) 3, (**F**) 6, or (**G**) 9 days of treatment. All data are presented as mean values ± SEM, combined from 2 independent experiments with 4 to 5 individually analyzed mice per group. Each symbol in scatter plots represents 1 individual mouse. Data were analyzed with Student’s t test and significance is indicated by the following symbols: * *p* < 0.05, ** *p* < 0.01, **** *p* < 0.0001 vs. WT.

In the first set of experiments, the efficacy of DT in inducing monocyte depletion was tested in mice without any additional surgical treatment. Injections (PBS for WT mice (Figure 2A), DT for CD11b-DTR mice (Figure 2B)) were administered every other day for a total of 3, 6, or 9 days (Figure 2C), mimicking phase-specific treatment in later experiments, followed by terminal blood collection. Next, a murine model of distal colonic anastomosis was established and performed in CD11b-DTR and WT mice. After surgery, phase-specific DT-induced monocyte and MΦ ablation was conducted during the inflammatory (days 0–3), proliferative (days 4–10), or reparative (days 11–20) phases of intestinal AH. On the postoperative day (POD) 3, 9, or 20, mice were sacrificed for tissue and blood examination to study phase-specific MΦ-associated changes in intestinal AH.

### 2.4. Surgical Procedure

All mice received buprenorphine (0.1 mg/kg; Temgesic^®^, Indivior, Berkshire, UK) subcutaneously for pre- and postoperative analgesia. Anesthesia was performed using a mixture of N_2_O/O_2_ = 2:1 (Westfalen AG, Münster, Germany) and 4% isoflurane (Forene^®^, AbbVie, Wiesbaden, Germany), and subsequently was maintained by a mixture of N_2_O/O_2_ = 2:1 and 1.5% isoflurane. Spontaneous breathing of mice was maintained during surgery and a body temperature of 37 °C was secured by a heating pad (ThermoLux, Murrhardt, Germany). For surgery, mice were fixated in a supine position and their fur in the surgical area was removed. After median laparotomy, the rectosigmoid junction was detected, and small bowel tissue was mobilized to the paracolic space. In the microsurgical technique, using an operating microscope (Carl Zeiss, Oberkochen, Germany), a transection of the distal colon was performed under preservation of the vasculature. A standardized end-to-end anastomosis was conducted (Supplementary Figure S1), consisting of 12 seromuscular single button stitches of non-absorbable monofilament ETHILON™ 9-0 (Polyamide 6,6, Ethicon^®^, Norderstedt, Germany). Anchor stitches were set next and opposite to the mesocolon, followed by 5 stitches on the anterior and after a 180° rotation of the tissue, 5 stitches on the posterior wall. The colon was constantly moisturized by preheated PBS. The peritoneum was closed by means of single button stitches of absorbable monofilament PDS*II 5-0 (Polydioxanone, Ethicon^®^, Ethicon, Norderstedt, Germany) and the skin was closed by means of single button stitches of absorbable braided Coated VICRYL™ 4-0 (Polyglactin 910, Ethicon^®^, Ethicon, Norderstedt, Germany). Post-operative care comprised an immediate supply of heat, water, and food.

### 2.5. Tissue Harvest and Evaluation of Anastomotic Bursting Pressure

At the end of the respective observation period, blood was extracted via cardiac puncture under general anesthesia and analgesia, followed by cervical dislocation. Tissue samples were rinsed and cleaned with cold PBS, then quick-frozen or stored in 4% phosphate-buffered formalin (Langenbrinck, Emmendingen, Germany). The anastomosis-bearing colon segment was carefully exposed to perform the anastomotic bursting pressure (ABP) test. Briefly, a measurement catheter (Neurovent-P, Raumedic^®^, Helmbrechts, Germany) connected through a pressure-cable (Raumedic^®^) to a Zero-Point Stimulator (NSP2; Raumedic^®^) was introduced aborally, whereas a perfusor line (B. Braun, Melsungen, Germany) was placed orally to the anastomosis. Both ends were tightly ligated with braided silk (Resorba^®^, Nürnberg, Germany), and natrium chloride 0.9% (B. Braun) was steadily infused (2 mL/min). The intraluminal pressure leading to tissue bursting was recorded. After testing the ABP, the anastomosis-carrying colon segment (±1 cm) was halved lengthwise, with one half fixated on a panel in formalin and the other one quick-frozen for further analysis. After preservation in paraffin (Histosec™ pastilles; Sigma-Aldrich), the tissue was cut in consecutive sections (5 µm), which were subjected to hematoxylin (Sigma-Aldrich) and eosin (Morphisto^®^, Offenbach am Main, Germany) or immunofluorescent staining.

### 2.6. Fluorescence-Activated Cell Sorting (FACS)

FACS analysis was used for the assessment of systemic monocyte ablation and was performed on full blood samples using 100 µL of whole blood per specimen. Whole blood was collected in lithium heparin tubes and immediately used for FACS staining. First, blood was incubated with 5 µg/mL Fc-block (TruStain FcX^TM^, Biolegend, San Diego, CA, USA) for 15 min at room temperature (RT; 20–22 °C) before 1 µg/mL antibodies were added as follows: anti-CD11b (M1/70)-BV421, anti-Ly6C (HK1.4)-APC, anti-Ly6G (1A8)-PE (all Biolegend, San Diego, CA, USA). After thorough vortexing, blood was incubated for 30 min at 4 °C. Then, 100 µL of one-step Fix/lyse solution was added to each tube and incubated for another 15 min at RT to allow for the lysis of erythrocytes and fixation of the stained cells. Tubes were filled with FACS buffer (PBS, 1% vol/vol BSA) and centrifuged at 350× *g* (RT) for 10 min. The supernatant was discarded, and cells were resuspended with 1 mL of FACS buffer and washed. After the final centrifugation, cells were resuspended in 300 µL of FACS buffer. Measurement was performed in Flow Cytometer CytoFlex S (Beckman Coulter, Krefeld, Germany), and the acquired data were analyzed using FloJo software 10.8.1. The relative abundance of monocytes (defined as CD11b^+^Ly6G^low^Ly6C^+^cells) in the blood was expressed as a percentage of CD11b+ cells.

### 2.7. Histological Analysis

Three nonconsecutive sections of the anastomosis were stained with hematoxylin and eosin (H&E) and analyzed by a board-certified and experienced pathologist, blinded to treatment modalities. The phase-specific AH was evaluated using a standardized histological anastomotic healing score (AHS) as previously described [24], including the following parameters: blood vessel ingrowth (0–4 points); fibroblasts (0–4 points); collagen formation (0–4 points), inflammatory cells (0–4 points, inversed); first layer in which continuity has been restored (counted from the mucosa outwards towards serosa; 0–4); the number of healed layers (0–4); epithelium closed (0 = no, 1 = yes); crypt architecture restored (0 = no, 1 = yes) and overall healing quality (1–3 points). In addition, quantification of the perianastomotic inflammatory state (defined as the severity of inflammatory cell infiltration) was conducted using a standardized semiquantitative anastomotic inflammation score (AIS) consisting of 4 parameters (0 = absent; 1 = mild; 2 = moderate, 3 = dense) as previously described [25]. Hypertrophic scar tissue (defined as granulation tissue outside the physiological colonic structure exclusive of ectopic organs and fat adhesions) and re-epithelialization (defined as the distance between epithelial edges) were measured with ImageJ (NIH, Bethesda, MD, USA). The collagen density of scar tissue was further investigated using picrosirius red (Morphisto^®^, Offenbach am Main, Germany) staining and the abundance of collagen I and III was expressed as a fraction of the scar area. The architecture of the colon in the perianastomotic region was further determined by measuring the thickness of the mucosa, submucosa, and muscularis on the oral and aboral edge using ImageJ.

### 2.8. Immunofluorescence (IF)

Three nonconsecutive sections of the anastomosis were treated as described above, then deparaffinized and rehydrated in a descending ethanol series. After heat-induced epitope retrieval with citrate concentrated solution (100×; Santa Cruz, Dallas, TX, USA) and Tween 20^®^ (Sigma), a blocking solution was applied for 1 h, containing 5% goat, 5% horse (VWR™ International GmbH, Darmstadt, Germany) and 5% fetal calf serum (Sigma) as well as 0.1% Triton^®^ X100 (Carl Roth^®^, Karlsruhe, Germany). The sections were then incubated overnight at 4 °C with primary antibodies against either F4/80 (1:100; Bio-Rad, Hercules, CA, USA) or CD31 (1:100; dianova, Eching, Germany). The sections were then stained for 1 h with anti-rat IgG2a secondary antibody (1:300; Novus, Wiesbaden, Germany), 4,6-diamidino-2-phenylindole (DAPI, 1:100,000; ThermoScientific™, Waltham, MA, USA) for nuclear marking and 5% goat, 5% horse and 5% fetal calf serum against the non-specific binding. After being washed in PBS, slides were mounted (Shandon™, Immu-Mount™, Epredia™, Kalamazoo, MI, USA). The local intestinal MΦ burden was analyzed in three pictures at ×10 magnification in three nonconsecutive slides per animal. First, the anastomosis-carrying colon segment (exclusive adhesions) was defined as a region of interest (ROI). Using automated cell counting software, adjusting the threshold, and selecting ROI individually, the number of MΦ, defined as F4/80-positive cells, was expressed in relation to the number of nuclei, defined as DAPI-positive cells. The perianastomotic neoangiogenesis was expressed as blood vessels per square millimeter (mm^2^). Using an ×20 magnification, three pictures of three nonconsecutive slides per animal were analyzed and blood vessels were defined as CD31 (PECAM)-positive structures with the lumen. Blood vessels were counted manually and expressed per mm^2^. All visualization and evaluation processes were realized with a BZ-X800 fluorescence microscope (Keyence, Neu-Insenburg, Germany) and its associated analyzer software.

### 2.9. Real-Time Quantitative PCR (qPCR)

Quick-frozen tissue was treated with RLT buffer (Qiagen, Venlo, The Netherlands) and 1% β-mercaptoethanol (Sigma), then homogenized and lysed (TissueLyser LT and stainless-steel beans 7 mm, Qiagen). Total RNA was extracted from the lysate, using the QiaCube protocol and AllPrep DNA/RNA/Protein Mini Kit (Qiagen). Additionally, the RNA material was treated with the Turbo DNA-free Kit (ThermoScientific™). The First Strand cDNA Synthesis Kit (ThermoScientific™) was used for cDNA synthesis according to the manufacturer’s instructions. The dilution for real-time qPCR was prepared by adding SsoAdvanced™ Universal SYBR^®^ Green Supermix (Bio-Rad) and primers (metabion international AG, Planegg/Steinkirchen, Germany): *EGF* (for 5′-CGG ACA GCT ACA CGG AAT G-3′, rev 5′-CGA GGC AGA CAC AAA TAA CCC-3′), *PDGF-A* (for 5′-GAT CCA CCT CGC ATC ATC TT-3′, rev 5′-GTT CCC GAC AGG AAA ACT CA-3′), *PDGF-B* (for 5′-GAT CTC TCG GAA CCT CAT CG-3′, rev 5′-GGC TTC TTT CGC ACA ATC TC-3′), *VEGF* (for 5′-GCT GTA ACG ATG AAG CCC TG-3′, rev 5′-TCG TCT TCT CAC CCT CAA CC-3′), *TGF-β1* (for 5′-AGA CAT CTC ACA CAG TAT-3′, rev 5′-CCA GGA ATT GTT GCT ATA-3′), eukaryotic translation elongation factor 2 (*Eef2*) (for 5′-TGT CAG TCA TCG CCC ATG TG-3′, rev 5′-CAT CCT TGC GAG TGT CAG TGA-3′). qPCR was realized using the CFX96™ Touch™ Real-Time System (Bio-Rad). The expression rates, normalized to the housekeeping gene *Eef2*, were evaluated by applying the 2^−∆∆Ct^ method.

### 2.10. Next Generation Sequencing and RNAseq Analysis

The library preparation of total RNA (see above) was performed with the NEBNext Ultra II RNA directional Kit and single read sequencing was performed using a NextSeq ^®^ 2000 System with a read length of 72 bp. The samples were demultiplexed with the Illumina^®^ DRAGEN™ Bio-IT Platform. Quality control was performed using FastQC version 0.11.9 [26]. Trimmomatic version 0.39 [27] was used for an adapter and low-quality end trimming as well as for general quality trimming utilizing a sliding window of 4 bp with a minimal average base quality of 15. Reads below a minimum read length of 15 bp were discarded. The resulting reads were aligned to the Ensembl GRCm39 reference genome using HISAT2 version 2.1.0 [28] and were sorted using SAMtools version 1.9 [29]. Gene-based read counting was performed using HTSeq version 0.12.4 [30] with the Ensembl annotation version 107. Ensembl IDs were converted to mgi symbols with the R package biomaRt [31]. Differential expression analysis was performed using the R package DESeq2 version 1.32.0 [32]. Gene set enrichment analysis (GSEA) was performed using the R package fgsea [33] with Gene Ontology (GO) terms from the Molecular Signatures Database (MSigDB) msigdbr [34]. For pathway analysis, the GO terms inflammatory response (GO:0006954), wound healing (GO:0042060), collagen metabolic process (GO:0032963), cytokine production (GO:0001816), and cell population proliferation (GO:0085029) were analyzed, including plotting of all direct descendants. A significance threshold of 0.05 was used for the FDR-corrected p-values to determine significantly expressed genes and gene sets. Heatmaps were created using the R package gplots [35] and scatterplots, volcano plots, and bar plots using ggplot2 [36]. A total of 422 genes (Supplementary Table S1) related to wound healing were identified from msigdbr using GO term 0042060 (“wound healing”); of these, the top 50 differently regulated (lowest *p* value) genes per timepoint were plotted as a heatmap.

### 2.11. Statistical Analysis

The purpose of this study was to test the null hypothesis that MΦ depletion would not interfere with phase-specific intestinal AH. The sample size was analyzed by G*Power analysis to ensure adequate power to detect a prespecified effect size to possibly reject the null hypothesis. All statistical analyses were performed using GraphPad Prism 9 (GraphPad Software, San Diego, CA, USA). Groups were compared using an unpaired two-tailored t-test, considering *p* < 0.05 as statistically significant. Results are presented as mean ± SEM and experiment-specific n values are indicated in the corresponding figure.

## 3. Results

### 3.1. The CD11b-DTR Mouse Is a Reliable Murine Model for Phase-Specific Systemic Monocyte and Colonic MΦ Depletion

Since intestinal MΦ are predominantly monocyte-derived, it was first tested whether the aforementioned phase-specific DT injection scheme (Figure 2B) would result in a reliable and reproducible systemic monocyte depletion in CD11b-DTR mice. Thus, WT and CD11b-DTR mice without surgical intervention were treated with PBS or DT, respectively, and sacrificed for blood collection after 3, 6, or 9 days of treatment. Figure 2C,D display exemplified analysis and gating strategies of the FACS analysis, which revealed that compared to PBS-treated WT mice (Figure 2C), i.p. DT injections in CD11b-DTR mice (Figure 2D) elicited a significant reduction in circulating monocytes (defined as CD11b^+^Ly6G^low^Ly6C^+^ cells), irrespective of the length of treatment. A significant reduction in the number of circulating monocytes was found after 3 (Figure 2C), 6 (Figure 2D), and 9 (Figure 2E) days of DT treatment when comparing WT and CD11b-DTR.

**Figure 3:**
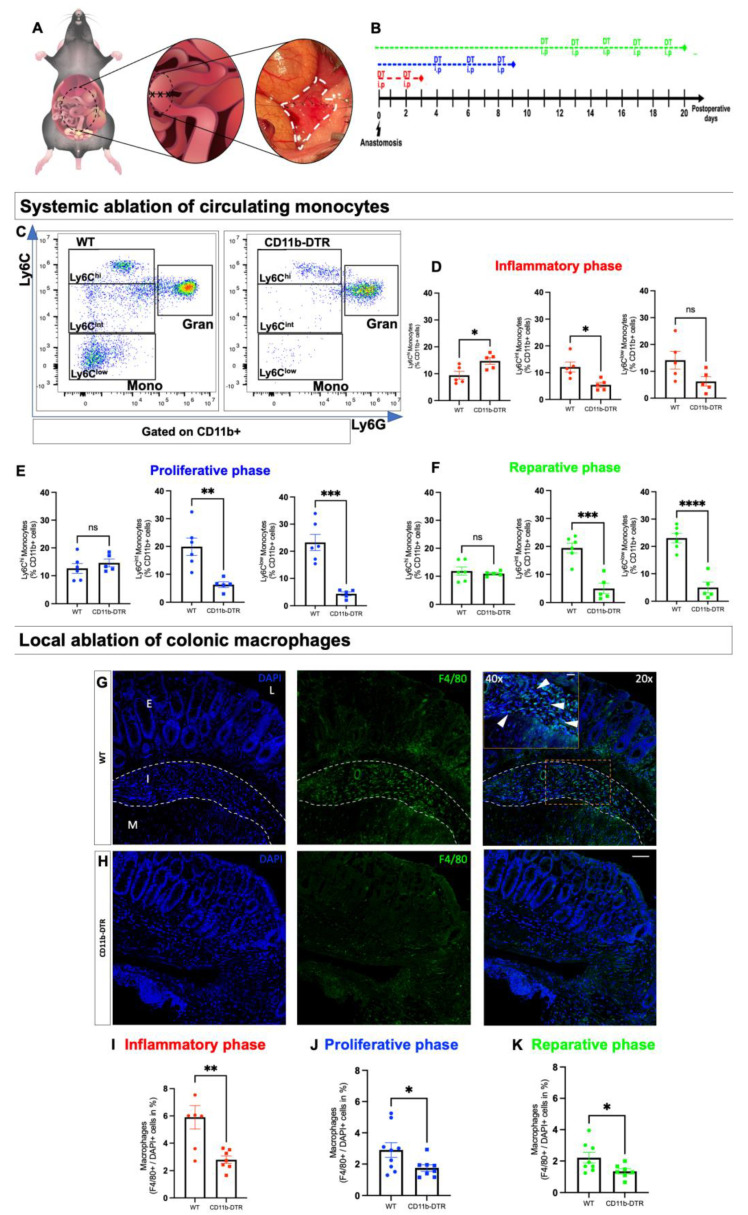
Diphtheria-toxin-induced circulating monocyte and intestinal macrophage depletion during intestinal anastomotic healing. (**A**) Colonic end-to-end anastomosis at the rectosigmoid junction as a model of murine intestinal anastomotic healing (AH) in wild type (WT) and CD11b-diphtheria toxin receptor (CD11b-DTR) mice. (**B**) Post-operative intraperitoneal (i.p) diphtheria toxin (DT) injective regime. After surgery, phase-specific DT-induced monocyte and macrophage ablation is conducted during the inflammatory (red, day 0–3), proliferative (blue, day 4–10), or reparative (green, day 11–20) of intestinal AH. On post-operative day (POD) 3, 9 or 20, mice are sacrificed for tissue and blood examination. (**C**) Representative dot plots (initially gated on viable CD11b cells) of circulating monocytes (CD11b^+^Ly6G^low^Ly6C^+^cells), stratified for Ly6C^high(hi)^, Ly6C^intermediate(int)^ and Ly6C^low^ subsets. Numeric data for circulating monocytes subsets in WT and CD11b-DTR mice during (**D**) the inflammatory, (**E**) the proliferative and (**F**) the reparative phase of intestinal anastomotic healing. Representative immunofluorescence images of F4/80-stained sections of the anastomotic region in WT (**G**) and (**H**) CD11b-DTR (**H**) mice. To better visualize the presence of macrophages (F4/80+), representative single color and merged pictures with DAPI (nuclei) staining are shown. All overview images were taken at 20× magnification, scale bar 50 µm, while close ups (dashed line) were taken at 40× magnification; scale bar 20 µm, white arrows indicate macrophages, L= lumen, I = perianastomotic infiltrate (outlined with white broken line), E = epithelium, M = muscularis. For quantitative analysis, 3 randomly chosen fields are analyzed on 3 non-consecutive slides. The intestinal macrophage burden is expressed as F4/80-stained cells per DAPI-stained cells in % and shown for the (**I**) inflammatory, (**J**) proliferative and (**K**) reparative phase of intestinal AH in WT and CD11b-DTR mice. All data are presented as mean values ± SEM, combined from at least 2 independent experiments with 5 to 9 individually analyzed mice per group. Each symbol in scatter plots represents 1 individual mouse; line indicates mean values. Data were analyzed with Student’s t test and significance is indicated by the following symbols: * *p* < 0.05, ** *p* < 0.01, *** *p* < 0.001, **** *p* < 0.0001 vs. WT, ns = not significant.

Having established a reliable protocol for on-demand phase-specific systemic monocyte reduction, a murine model of distal colorectal anastomosis (Figure 3A) was set up in both CD11b-DTR and WT mice. The study animals were divided into three groups to investigate the impact of phase-specific systemic monocyte and local colonic MΦ depletion during the three phases of intestinal AH. After surgery, phase-specific DT-induced monocyte and MΦ ablation was conducted (Figure 3B) during the inflammatory (day 0–3), proliferative (day 4–10), or reparative (day 11–20) phase of intestinal AH. Mice were sacrificed on postoperative days (POD) 3, 9, or 20 for tissue and blood examination. FACS analysis was performed in WT and CD11b-DTR mice to confirm systemic monocytopenia. It was confirmed that DT treatment induced a significant reduction in circulating monocytes in CD11b-DTR mice compared to WT mice. To further characterize the impact of DT treatment on different monocyte subsets during AH, CD11b^+^Ly6G^low^Ly6C^+^ cells were further classified based on the expression of Ly6C and stratified as Ly6C^hi^, Ly6C^int^, or Ly6C^low^ monocytes (Figure 3C). It was found that phase-specific DT treatment during the inflammatory phase of AH resulted in a significant increase in the frequency of Ly6C^hi^ in CD11b-DTR mice, while the number of Ly6C^int^ monocytes was significantly reduced (Figure 3D). The frequencies of Ly6C^low^ monocytes were comparable between WT and CD1b-DTR mice (Figure 3D). During the proliferative (Figure 3E) and reparative phase (Figure 3F), DT treatment elicited a significant reduction in the frequency of Ly6C^int^ and Ly6C^low^ monocytes, while the frequencies of Ly6C^hi^ monocytes were comparable between WT and CD11b-DTR mice.

Next, the impact of DT treatment on local intestinal MΦ burden in CD11b-DTR mice during AH was investigated. Cross-sections of anastomosis-carrying colon segments were used for IF staining against the F4/80 antigen, an established MΦ marker. In WT mice, intestinal MΦ were predominantly found in the densely populated perianastomotic immune cell infiltrate, but also in close proximity to the epithelium as well as in the submucosa (Figure 3G). In contrast, DT-treated CD11b-DTR mice displayed a marked reduction in intestinal MΦ in all regions (Figure 3H). This result demonstrating a significant DT-induced local MΦ reduction (expressed as % of DAPI positive cells) was consistent throughout all phases of AH and was found during the inflammatory (Figure 3I), proliferative (Figure 3J), and reparative (Figure 3K) phases.

### 3.2. A Reduced Burden of MΦ in CD11b-DTR Attenuates AH-Elicited Inflammation

**Figure 4:**
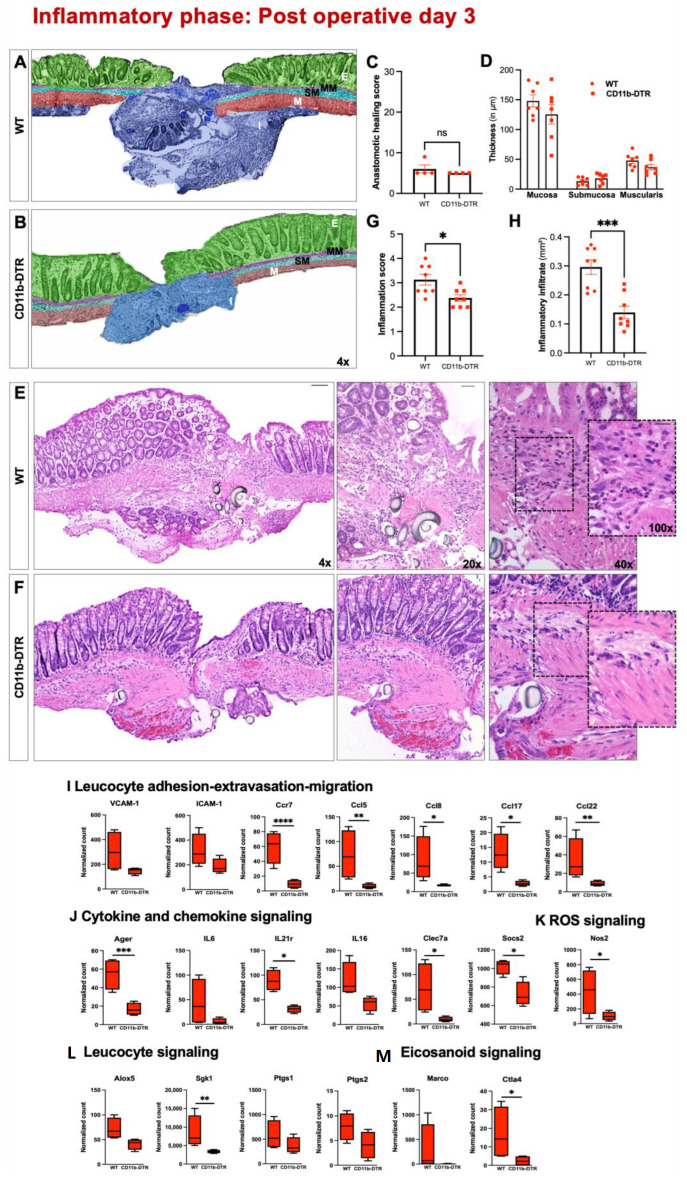
Intestinal anastomotic healing during the inflammatory phase. Multicolor stained sections of anastomotic healing (AH) at postoperative day 3 in (**A**) wild type (WT) and (**B**) CD11b-diphtheria toxin receptor (DTR) mice. E = epithelium (green); MM = muscularis mucosae (purple); SM = submucosa; M, tunica muscularis (turquoise); M = muscularis (red), I = perianastomotic infiltrate. Images were taken at 4× magnification, scale bar 100 µm. (**C**) Anastomotic healing score. (**D**) Thickness of colonic layers in the perianastomotic region (in µm). Representative histopathologic images of hematoxylin-and-eosin-stained anastomotic regions from (**E**) WT and (**F**) CD11b-DTR mice. Overview images were taken at 4× magnification (scale bar 100 µm), close-ups at 20× magnification (scale bar 50 µm), 40× magnification (scale bar 20 µm) and 100× magnification (scale bar 20 µm)). (**G**) Inflammatory infiltrate (in mm^2^) and (**H**) quantitative inflammation score for WT and CD11b-DTR mice. For quantitative analysis, 3 randomly chosen fields are analyzed per slide. Relative expression levels (normalized counts) of representative genes involved in (**I**) leucocyte adhesion-extravasation-migration, (**J**) cytokine and chemokine signaling, (**K**) reactive oxygen species (ROS) signaling, (**L**) leucocyte signaling and (**M**) eicosanoid signaling. All data are presented as mean values ± SEM, combined from at least 2 independent experiments with 4 to 8 individually analyzed mice per group. Each symbol in scatter plots represents 1 individual mouse; line indicates mean values. Data were analyzed with Student’s t test and significance is indicated by the following symbols: * *p* < 0.05, ** *p* < 0.01, *** *p* <0.001, **** *p* <0.0001 vs. WT, ns = not significant.

Having established a reliable protocol for on-demand and phase-specific systemic monocyte and local intestinal MΦ reduction as well as an animal model of distal colorectal anastomosis, we next aimed to further investigate the phase-specific role of MΦ during intestinal AH. Histological analysis of AH in WT and CD11b-DTR mice at POD 3 (inflammatory phase) revealed preliminary healing with no continuity in the bowel wall in both WT (Figure 4A) and CD11b-DTR (Figure 4B) mice. When quantifying AH by using the established AHS, no differences were found between groups (Figure 4C). In addition, the colon wall architecture remained comparable between WT and CD11b-DTR mice when measuring the thickness of the mucosa, submucosa, and muscularis (Figure 4D). However, when analyzing H&E-stained cross sections of the anastomotic region, it became evident that WT mice (Figure 4E) revealed a more pronounced inflammatory perianastomic infiltrate when compared to CD11b-DTR mice (Figure 4F). These findings were confirmed and quantified by measuring the perianastomic infiltrate (Figure 4G), as well as applying a standardized AIS (Figure 4H). Lastly, the observed histological evidence for a reduced inflammatory response in CD11b-DTR mice was confirmed at the genetic level. When comparing RNAseq data from anastomotic tissue of WT and CD11b-DTR mice, a marked upregulation of representative genes was found to be involved in hallmark events of inflammation, including leucocyte adhesion and extravasation (Figure 4I; *VCAM-1*, *ICAM-1*, *Ccr7*, *Ccl5*, *Ccl8*, *Ccl17*, *CCl22*), cytokine and chemokine signaling (Figure 4J; *Ager*, *IL6*, *IL21r*, *IL16*, *Clec7a*, *Socs2*), ROS signaling (Figure 4K: *Nos2*), leucocyte signaling (Figure 4L; *Alox5*, *Sgk1*, *Ptgs1*, *Ptgs2*) and eicosanoid signaling (Figure 4M; *Marco*, *Ctla4*) in WT mice.

**Figure 5:**
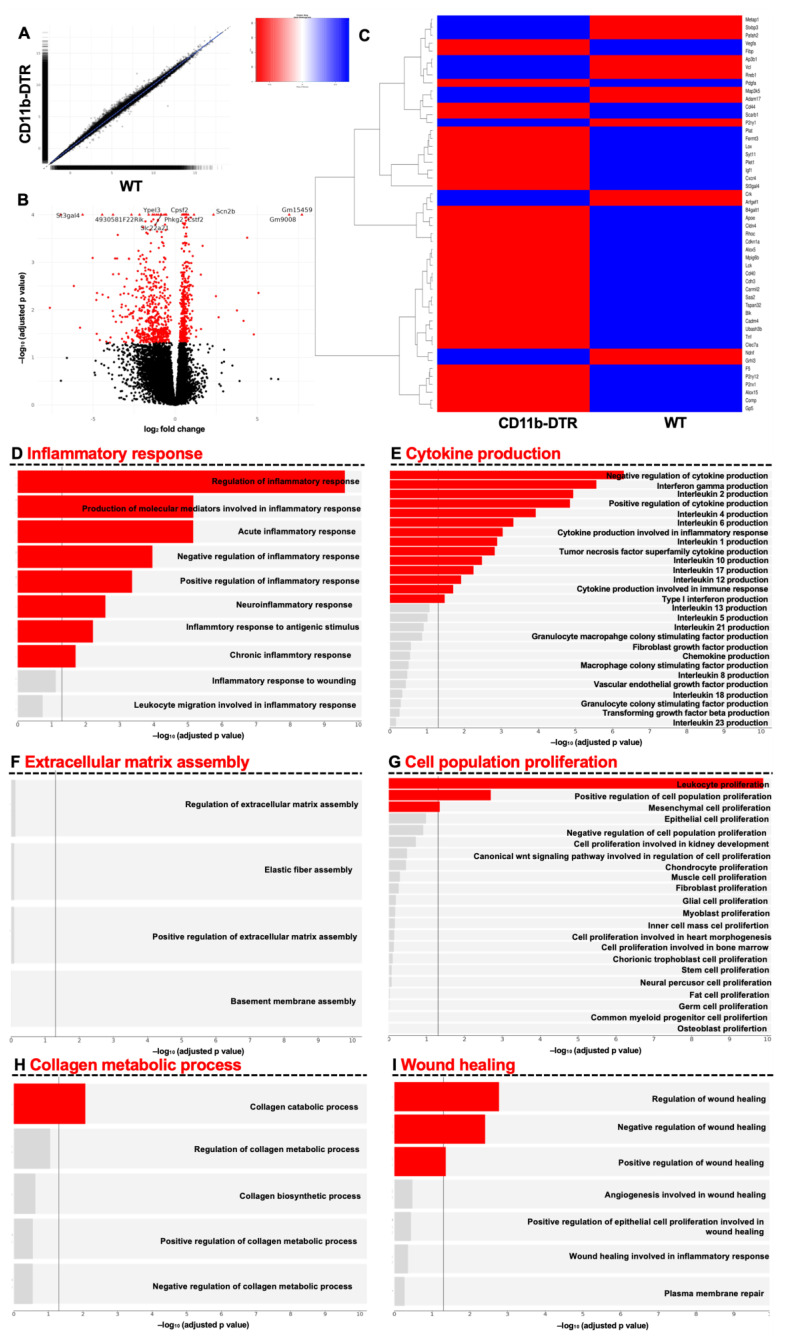
Gene set enrichment analysis of intestinal anastomotic healing during the inflammatory phase. Gene set enrichment analysis (GSEA) results of anastomotic healing (AH) comparing wild type (WT) with CD11b-diphtheria toxin receptor (DTR) mice. RNA-Seq was performed on samples collected at postoperative day 3. (**A**) Scatterplot and (**B**) volcano plot. Volcano plot showing statistical significance of differential gene expression data (adjusted *p*-value) vs. magnitude of expression change (log2 fold change). (**C**) Heatmap of the top 50 differentially regulated genes related to wound healing, identified using GO term 0042060 (“wound healing”). (**D**) Pathways under inflammatory response (GO:0006954), (**E**) pathways under cytokine production (GO:0001816), (**F**) pathways under extracellular matrix assembly (GO:0085029), (**G**) pathways under cell population proliferation (GO:0085029), (**H**) pathways under collagen metabolic process (GO:0032963) and (**I**) pathways under wound healing (GO:0042060). A significance threshold of 0.05 was used for the FDR-corrected p-values to determine significantly expressed genes and gene sets. Bars in red indicate statistically significant upregulation. A total of 4 mice were analyzed per group.

To further explore supraordinate regulation mechanisms, GSEA was used to identify differentially regulated pathways in WT and CD11b-DTR mice during the inflammatory phase of intestinal AH. Volcanos and scatterplots show differentially regulated genes between the groups at POD 3 (Figure 5A,B). It was found that a total of 981 genes were regulated significantly differently between WT and CD11b-DTR mice. Of these, a total of 419 genes were up- and 562 were downregulated. Next, a heatmap was used to compare the top 50 differentially regulated genes (Supplementary Table S2) involved in wound healing (GO term 0042060 (“wound healing”)) between WT and CD11b-DTR mice (Figure 5C). To further understand the alteration of pathways in WT and CD11b-DTR mice during the inflammatory phase of AH, specific supraordinate biological processes and their respective direct descendants were analyzed. It was found that WT mice displayed a significant upregulation in genes encoding for inflammatory responses (including the production of molecular mediators involved in inflammatory response) (Figure 5D). Compared to CD11b-DTR mice, WT mice demonstrated a significant upregulation of gene sets involved in cytokine production (including *IFN-y*, *IL-2*, and *IL-4*) (Figure 5E) as well as leukocyte proliferation (Figure 5G). Moreover, WT mice had significantly upregulated pathways involved in collagen metabolic production (Figure 5H) and wound healing (Figure 5I). No differences were found for ECM assembly during this early timepoint of intestinal AH (Figure 5F).

### 3.3. MΦ Depletion during the Proliferative Phase Results in Accelerated AH

**Figure 6:**
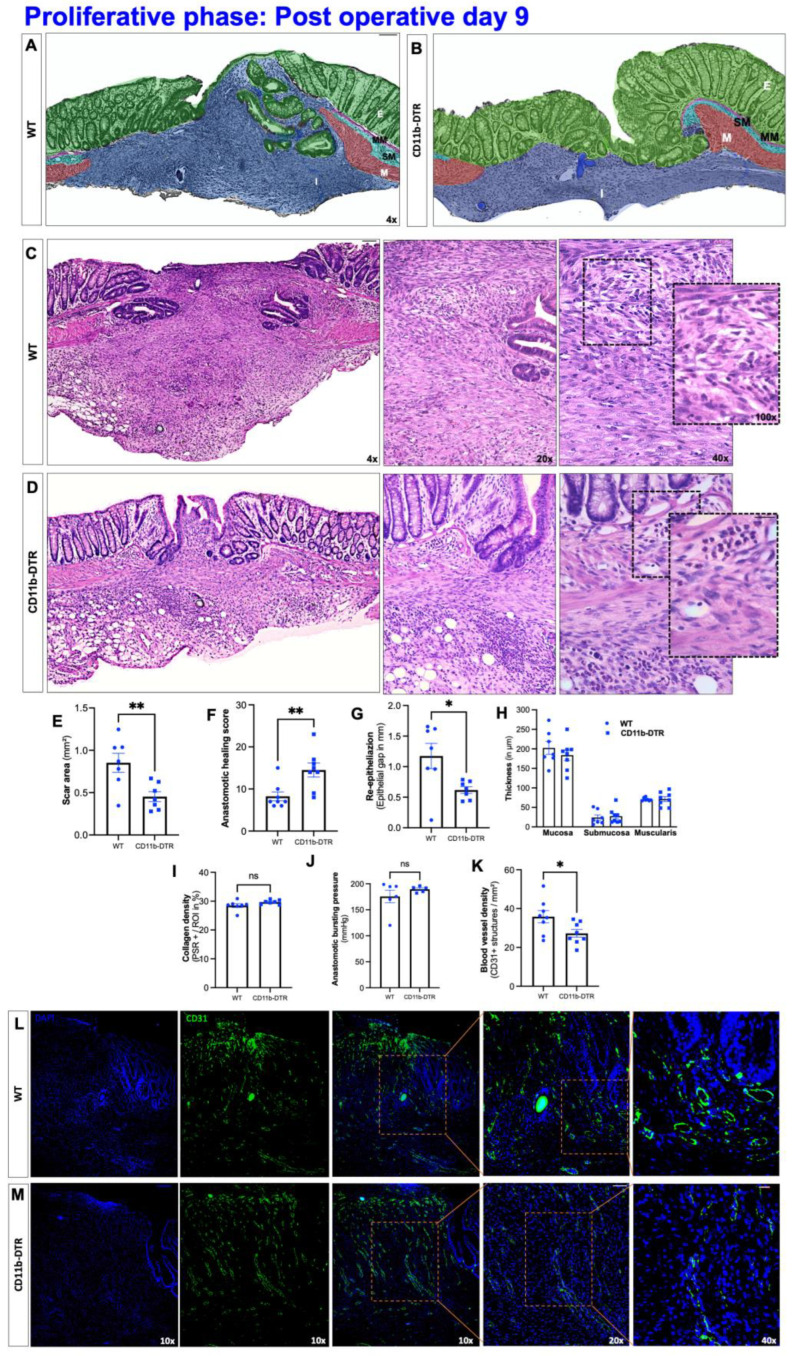
Intestinal anastomotic healing during the proliferative phase. Multicolor stained sections of anastomotic healing (AH) at postoperative day 9 in (**A**) wild type (WT) and (**B**) CD11b-diphtheria toxin receptor (DTR) mice. E = epithelium (green); MM = muscularis mucosae (purple); SM = submucosa; M, tunica muscularis (turquoise); M = muscularis (red), I = perianastomotic infiltrate. Images were taken at 4× magnification, scale bar 100 µm. Representative histopathologic images of hematoxylin-and-eosin-stained anastomotic regions from (**C**) WT and (**D**) CD11b-DTR mice. Overview images were taken at 4× magnification (scale bar 100 µm), close-ups at 20× magnification (scale bar 50 µm), 40× magnification (scale bar 20 µm) and 100× magnification (scale bar 20 µm)). (**E**) Scar area (in mm^2^), (**F**) anastomotic healing score, (**G**) re-epithelialization (epithelial gap in mm), (**H**) thickness of colonic layers in the perianastomotic region (in µm), (**I**) collagen density (picro-sirius red stained collagen (PSR+)/scar area) (**J**) anastomotic bursting pressure (in mmHg) and (**K**) blood vessel density (CD31+ structures/mm^2^). Representative immunofluorescence images of CD31-stained sections of anastomotic regions from WT (**L**) and CD11b-DTR (**M**) mice. To visualize blood vessels (CD 31+), representative single color and merged pictures with DAPI (nuclei) staining are presented. For orientation, images were taken at ×10 magnification (scale bar 100 µm), then orange marked section is magnified ×20 (scale bar 50 µm) and ×40 (scale bar 20 µm), and 4 consecutive chosen fields were analyzed in 3 non-consecutive slides. All data are presented as mean values ± SEM, combined from at least 2 independent experiments with 5 to 8 individually analyzed mice per group. Data were analyzed with Student’s t test and significance is indicated by the following symbols: * *p* < 0.05, ** *p* < 0.01, vs. WT, ns = not significant.

When AH at POD 9 (proliferative phase) was investigated, histological analysis revealed an overall progression of wound healing, with the start of bowel wall continuity as well as partially restored crypt architecture in both WT (Figure 6A) and CD11b-DTR (Figure 6B) mice. However, similar to POD 3, CD11b-DTR mice displayed a markedly reduced perianastomotic infiltrate, representing granulation tissue (Figure 6C–E). In addition, CD11b-DTR mice had a significantly higher AHS (meaning improved AH healing) when compared to WT mice (Figure 6F). The improved AH in DT-treated CD11b-DTR mice was also evidenced by the significantly accelerated re-epithelization (Figure 6G), while the thickness of each bowel layer remained comparable between WT and CD11b-DTR mice (Figure 6H). Since the proliferative phase of AH is characterized by a tightly organized balance between collagen degradation and synthesis (both of which are directed by MΦ), it was next investigated whether the collagen density within the granulation tissue would differ between CD11b-DTR and WT mice. As shown in Figure 6I, no significant differences were observed. To test whether the histological evidence for an enhanced AH would translate into a functional benefit, the ABP was tested, but no difference between CD11b-DTR and WT mice was noted (Figure 6J).

Since MΦ are key drivers of AH-associated blood vessel ingrowth via the secretion of pro-angiogenic growth factors, neoangiogenesis was compared between WT (Figure 6K) and CD11b-DTR (Figure 6L) mice by using IF. It was found that CD11b-DTR mice had a significantly reduced number of blood vessels in CD31-stained cross-sections, compared with WT mice (Figure 6M).

**Figure 7:**
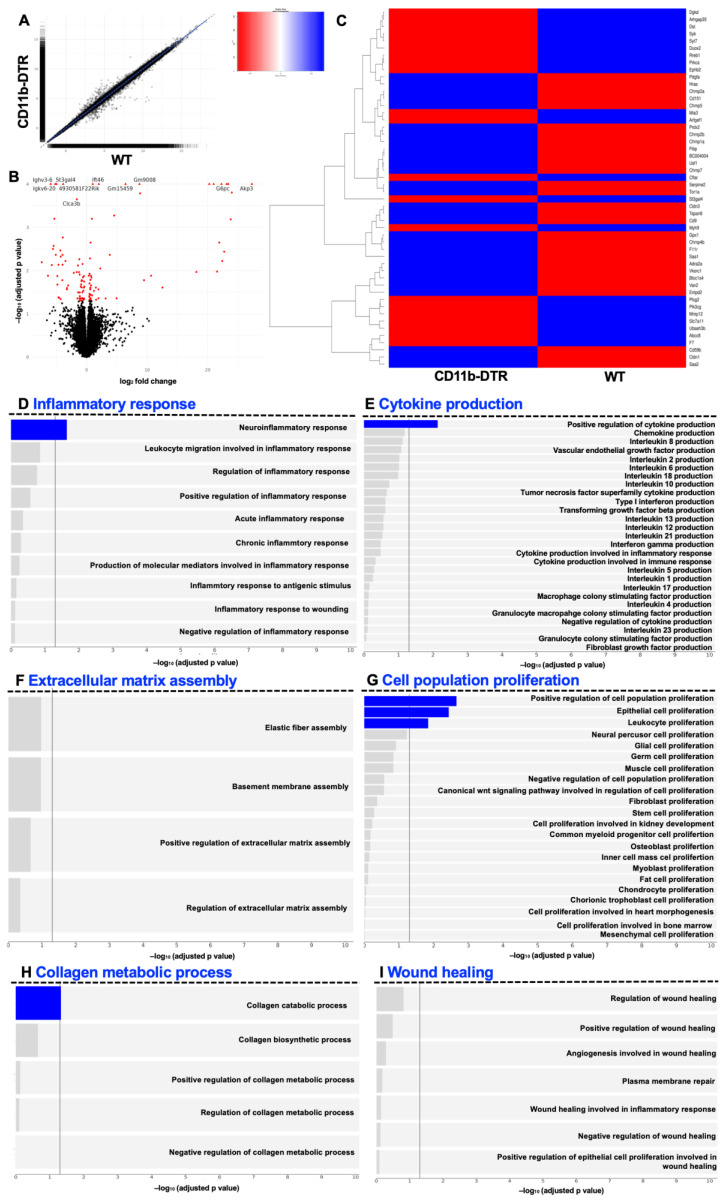
Gene set enrichment analysis of intestinal anastomotic healing during the proliferative phase. Gene set enrichment analysis (GSEA) results of anastomotic healing (AH) comparing wild type (WT) with CD11b-diphtheria toxin receptor (DTR) mice. RNA-Seq was performed on samples collected at postoperative day 9. Scatterplot (**A**) and volcano plot (**B**). Volcano plot showing statistical significance of differential gene expression data (adjusted *p*-value) vs. magnitude of expression change (log2 fold change). (**C**) Heatmap of the top 50 differentially regulated genes related to wound healing, identified using GO term 0042060 (“wound healing”). (**D**) Pathways under inflammatory response (GO:0006954), (**E**) pathways under cytokine production (GO:0001816), (**F**) pathways under extracellular matrix assembly (GO:0085029), (**G**) pathways under cell population proliferation (GO:0085029), (**H**) pathways under collagen metabolic process (GO:0032963) and (**I**) pathways under wound healing (GO:0042060). A significance threshold of 0.05 was used for the FDR-corrected p-values to determine significantly expressed genes and gene sets. Bars in blue indicate statistically significant upregulation. A total of 4 mice were analyzed per group.

Next, the GSEA was conducted to analyze WT and CD11b-DTR mice at POD 9. Volcano plots and scatterplots show differentially regulated genes between the groups at POD 9 (Figure 7A,B). It was found that a total of 125 genes were regulated significantly differently between WT and CD11b-DTR mice. Of these, 57 genes were up- and 68 were downregulated. Next, a heatmap was used to compare the top 50 differentially regulated genes (Supplementary Table S2) involved in wound healing (GO term 0042060 (“wound healing”)) between WT and CD11b-DTR mice (Figure 7C). Pathway analysis revealed only a slight but still significant upregulation in genes involved in inflammatory responses (Figure 7D) or cytokine production (Figure 7E) in WT mice. Of interest, a significant upregulation in gene sets encoding for epithelial proliferation (Figure 7G) and collagen catabolic processes (Figure 7H) was observed in WT mice.

### 3.4. Mediators Associated with Wound Reparation were Not Affected by MΦ Depletion

**Figure 8:**
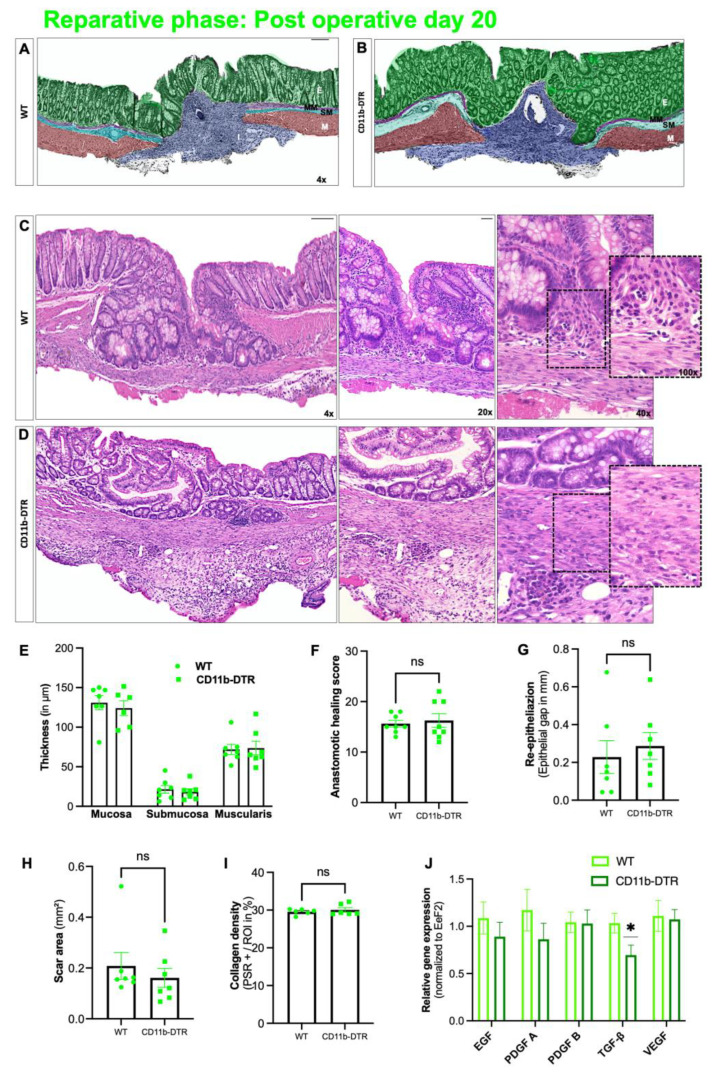
Intestinal anastomotic healing during the reparative phase. Multicolor stained sections of anastomotic healing (AH) at postoperative day 20 in (**A**) wild type (WT) and (**B**) CD11b-diphtheria toxin receptor (DTR) mice. E = epithelium (green); MM = muscularis mucosae (purple); SM = submucosa; M, tunica muscularis (turquoise); M = muscularis (red), I = perianastomotic infiltrate. Images were taken at 4× magnification, scale bar 100 µm. Representative histopathologic images of hematoxylin-and-eosin-stained anastomotic regions from (**C**) WT and (**D**) CD11b-DTR mice. Overview images were taken at 4x magnification (scale bar 100 µm), close-ups at 20× magnification (scale bar 50 µm), 40× magnification (scale bar 20 µm) and 100× magnification (scale bar 20 µm)). (**E**) Thickness of colonic layers in the perianastomotic region (in µm). (**F**) Anastomotic healing score, (**G**) re-epithelialization (epithelial gap in mm), (**H**) scar area (in mm^2^) and (**I**) collagen density. (**J**) Quantitative PCR analysis of results of macrophage derived growth factors (EGF: epidermal growth factor, PDGF-A/B: platelet-derived growth factor A/B, VEGF: vascular endothelial growth factor, *TGF-β1*: transforming growth factor beta 1) normalized to eukaryotic translation elongation factor 2 (Eef2) and analyzed by the 2^−∆∆Ct^ method. All data are presented as mean values ± SEM, with 5 to 8 individually analyzed mice per group. Each symbol in the scatter plots represents 1 individual mouse; line indicates mean values. Significance, analyzed with Student’s *t* test, compared with WT mice is indicated by the following symbols: * *p* < 0.05, ns = non-significant vs. WT.

Lastly, AH was investigated at POD 20 (reparative phase). Overall, WT (Figure 8A) and CD11b-DTR (Figure 8B) mice displayed well-progressed healing with bowel wall continuity and a closed epithelium (Figure 8C–E). Accordingly, AHS revealed no differences in microscopic AH (Figure 8F) and re-epithelization (Figure 8G) between the groups. Since the reparative phase is characterized by a continuous MΦ elicited remodeling of the granulation tissue, scar area (Figure 8H) as well as collagen density (Figure 8I) were determined. However, no significant differences were found between WT and CD11b-DTR mice. Next, qPCR was used to measure the expression of well-established MΦ-derived growth factors. While no differences were found for *EGF*, *PDGF A*, *PDGF B*, and *VEGF*, a significant downregulation of *TGF-β* in anastomotic samples of CD11b-DTR mice was revealed (Figure 8J).

**Figure 9:**
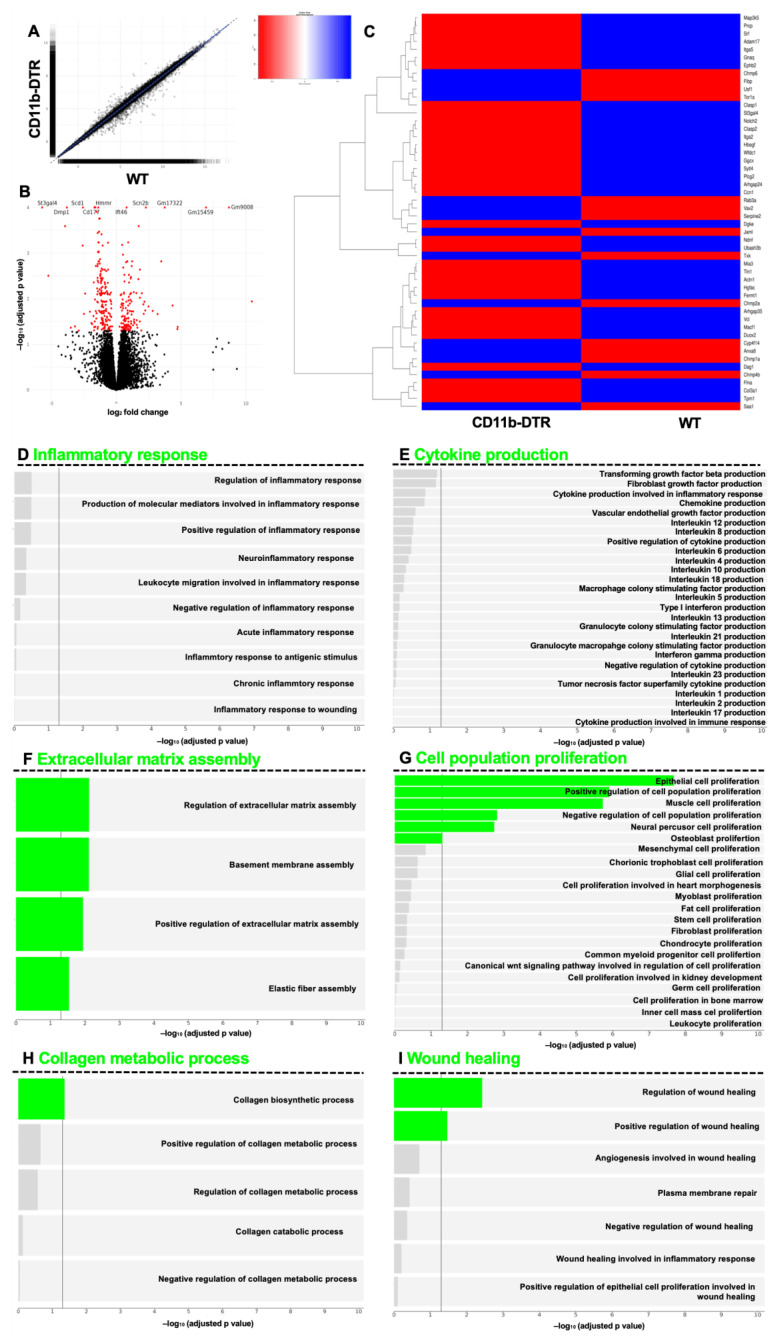
Gene set enrichment analysis of intestinal anastomotic healing during the reparative phase. Gene set enrichment analysis (GSEA) results of anastomotic healing (AH) comparing wild type (WT) with CD11b-diphtheria toxin receptor (DTR) mice. RNA-Seq was performed on samples collected at postoperative day 9. Scatterplot (**A**) and volcano plot (**B**). Volcano plot showing statistical significance of differential gene expression data (adjusted p-value) vs. magnitude of expression change (log2 fold change). (**C**) Heatmap of the top 50 differentially regulated genes related to wound healing, identified using GO term 0042060 (“wound healing”). (**D**) Pathways under inflammatory response (GO:0006954), (**E**) pathways under cytokine production (GO:0001816), (**F**) pathways under extracellular matrix assembly (GO:0085029), (**G**) pathways under cell population proliferation (GO:0085029), (**H**) pathways under collagen metabolic process (GO:0032963) and (**I**) pathways under wound healing (GO:0042060). A significance threshold of 0.05 was used for the FDR-corrected *p*-values to determine significantly expressed genes and gene sets. Bars in green indicate statistically significant upregulation. A total of 4 mice were analyzed per group.

Next, RNAseq analysis was conducted, and volcano and scatterplots show differentially regulated genes between the groups at POD 20 (Figure 9A,B). It was found that a total of 315 genes were regulated significantly differently between WT and CD11b-DTR mice. Of these, 138 genes were up- and 177 were downregulated. Next, a heatmap was used to compare the top 50 differentially regulated genes (Supplementary Table S2) involved in wound healing (GO term 0042060 (“wound healing”)) between WT and CD11b-DTR mice (Figure 9C). GSEA revealed no significant differences in gene sets involved in inflammatory responses (Figure 9D) or cytokine production (Figure 9E) in WT mice compared to CD11b-DTR. Of interest, significant differences were found for pathways encoding for the regulation of ECM assembly, basement membrane assembly, and elastic fiber assembly (Figure 9F). In line with this, pathway analysis revealed a significant upregulation of genes involved in epithelial cell proliferation, muscle cell proliferation, and neural precursor cell proliferation (Figure 9G), as well as collagen biosynthetic processes (Figure 9H) and wound healing (Figure 9I) in WT mice, when compared to CD11b-DTR mice.

## 4. Discussion

This study provides evidence demonstrating that intestinal MΦ elicit phase-specific key tasks during colonic AH and that temporal MΦ depletion attenuates pro-inflammatory as well as pro-resolving pathways during intestinal AH. It is convincingly demonstrated that in the early postoperative phase, intestinal MΦ greatly contribute to the inflammatory response, collagen hypertrophy as well as neoangiogenesis in the perianastomotic scar region and that MΦ depletion significantly improves microscopic AH. In the later postoperative phase, MΦ constitute a source of growth factors and are critically linked to tissue repair including ECM assembly and cell proliferation; however, later MΦ depletion had no effect on microscopic AH.

Intestinal wound healing is a complex process that is essential for the regeneration of epithelial integrity, tissue architecture, and homeostasis, tightly orchestrated by local and recruited immune cells. MΦ play a pivotal role throughout the cascade of inflammatory and resolving events, which has been addressed in multiple studies in various tissues, in particular experimental models of skin wound healing [37]. Although many of these findings can be applied to the intestine, the aspects of the individual microbiome and gut mobility complicate intestinal AH. In addition, MΦ function is now recognized as a result of spatiotemporal cues received from the local microenvironment, and thus, MΦ-associated pathways in dermal and intestinal wound healing are identified to be different. From an experimental perspective, it is also important to highlight that the underlying mechanisms responsible for undisrupted intestinal AH and wound healing in cutaneous punch biopsy wounds are markedly different, as the latter highly depends on wound contraction. Moreover, collagen subtypes and the cells contributing to collagen formation differ between the skin and the intestine. In addition, the inflammatory response during intestinal AH is of greater importance than it is for the skin [38]. Accordingly, one of the most important aspects is that impaired wound morphology and delayed healing during intestinal AH have a dramatic functional consequence with a recognized clinical feature, namely AL, which is in broad contrast to complications of impaired cutaneous healing including transformation into a chronic wound or infection. Therefore, additional research on intestinal AH is of paramount importance.

The current results are partially in contrast to previous findings obtained in murine models of dermal wound healing. Using CD11b-DTR mice, Mirza et al. found that MΦ depletion resulted in delayed re-epithelialization, decreased collagen deposition, reduced angiogenesis, diminished cell proliferation as well as increased levels of TNF-α and reduced levels of growth factors [39]. However, in contrast to the phase-specific depletion strategy used in the present study, Mirza et al. depleted MΦ at the time of wounding as well as 48 h later. Goren et al. used a lysozyme M promoter-driven and Cre-mediated DTR strategy and revealed that MΦ depletion was associated with impaired skin wound healing and reduced neoangiogenesis. Of interest, they also demonstrated an exacerbated inflammatory response in MΦ depleted mice [40]. Lucas et al. used a similar model but were the first to specifically study phase-dependent MΦ depletion during skin repair [41]. Their data revealed that MΦ depletion during the inflammatory phase diminished epithelialization and reduced the formation of granulation tissue. During the repair phase, MΦ depletion resulted in impaired angiogenesis, while a later MΦ depletion in the healing process had no further impact on wound repair, which is in line with the current results. In summary, the model of intestinal AH used here and the mentioned models of skin repair have all demonstrated reduced scar formation as well as attenuated angiogenesis following MΦ depletion, while demonstrating conflicting results regarding the inflammatory state following wounding in MΦ-depleted mice.

The currently available data regarding the role of MΦ during intestinal AH are scarce. Wu et al. found the presence of CD206+ anti-inflammatory MΦ to be associated with reduced rates of AL in a murine model of AH during peritonitis [42]. Cetinkaya et al. reported that granulocyte macrophage-colony stimulating factor (GM-CSF) improved AH complicated by intraperitoneal mitomycin-C application [43]. On the contrary, Pantelis et al. could demonstrate that genetic or pharmacological (using clodronate) muscularis MΦ depletion did not influence ileal or colonic AH, even when complicated by endotoxemia. However, there are crucial differences in the present study. This study is the first to specifically deplete MΦ during the sequential phases of intestinal AH. In addition, the use of a DT-based on-demand depletion strategy circumvents several problems that arise from conventional knock-out strategies. Furthermore, this study provides in-depth GSEA, which further helps to overcome the problem of morphological descriptions.

The distinct role of MΦ during the inflammatory phase of AH remains elusive [44]. Some studies suggest that MΦ depletion diminishes phagocytosis of apoptotic cells, bacteria, and foreign debris, leading to prolonged damage-associated molecular patterns (DAMPS, e.g., high-mobility group Box 1 protein (HMGB-1)) release, and thus causing an amplified inflammatory response [45]. Accordingly, Goren et al. and Mirza et al. found increased levels of TNF-α and a heightened neutrophil presence in skin wounds after MΦ depletion. The initial inflammatory response is also crucial for subsequent AL since inflammatory monocytes and MΦ produce MMP-2 and -9, which has been recently shown to be associated with AL in a murine model of colonic anastomosis [25]. However, the results of significantly reduced microscopic inflammation as well as the identification of MΦ-dependent inflammatory gene sets are in line with previous reports suggesting that tissue-resident MΦ be crucially involved in neutrophil recruitment and the initiation of the early inflammatory response [46]. This is further supported by the finding of the reduced expression of genes involved in leucocyte recruitment as well as cytokine and chemokine signaling in CD11b-DTR mice. The reduced inflammatory state (in contrast to previous reports) could be explained by the used DT treatment strategy, which depleted tissue-resident MΦ, thereby blocking the initiation of inflammation as well as circulating monocytes, blocking the subsequent monocyte influx. The data presented here also suggest that despite the significant MΦ depletion, the remaining mucosal immune system in the used model is sufficient to foster an immunological response to the tissue trauma during AH. This is of critical importance, since bacteria in particular are involved in collagen degradation and subsequent AL [47].

Besides sufficient microscopic wound healing, intestinal AH aims to provide anastomotic strength, which is directly associated with collagen metabolism. One common finding in wound healing models coupled with MΦ depletion is that of reduced collagen deposition and granulation tissue formation. The data extend this concept to intestinal AH, demonstrating collagen metabolic processes and extracellular matrix assembly to be highly MΦ-dependent. Although a significantly reduced amount of granulation tissue was found in CD11b-DTR mice, the respective collagen density remained comparable to WT mice. In addition, the breaking strength of colorectal anastomoses was also comparable between CD11b-DTR and WT mice. This points to an apparent discrepancy between the enhanced microscopic AH (improved histological healing, reduced scar area, diminished perianastomotic infiltrate) and the comparable ABP between WT and CD11b-DTR mice at POD9. One possible explanation is that the concept of phase-specific AH is temporally dynamic, and the defined endpoints at POD 3, 9 and 20 may not fully recapitulate all features of the respective phase. Therefore, an improved microscopic AH at POD9 may not directly translate into an improved mechanical stability, or would require later testing, e.g., at POD12 or 15. In line with this, further studies are needed to better define the time frame of the inflammatory, proliferative, and reparative phase of intestinal AH.

Since intestinal MΦ are predominately replenished by a constant monocyte influx (especially under inflammatory conditions, e.g., AH), it is paramount to not only deplete intestinal MΦ but also circulating monocytes when establishing a temporally selective and cell type-specific depletion strategy during intestinal AH. There are three well-defined monocyte subsets in humans when stratified by the expression of CD14 and CD16 surface antigens: classical (CD14^++^CD16^−^), intermediate (CD14^+^CD16^+^), and nonclassical (CD14^dim^CD16^+^) monocytes [48]. Analogically, murine monocytes are divided based on lymphocyte antigen 6 complex expression, locus C (Ly6C), and can be grouped according to their human counterparts: Ly6C^hi^ (Ly6^+^, classical or inflammatory), Ly6C^int^ (Ly6^+^, intermediate) and Ly6C^low^ (Ly6^-^, non-classical) [49,50]. Notably, traditionally, Ly6C^+^ monocytes were seen as the main monocyte population to enter the gut; however, there is emerging evidence that suggests a crucial role of intermediate and non-classical monocytes in inflammatory conditions, including wound healing [51]. Ly6C^hi^ monocytes were shown to be more dominant in the early inflammatory phase, exhibiting phagocytic and inflammatory functions, whereas Ly6C^low^ monocytes dominate the later phase, displaying anti-inflammatory properties and promoting healing [52]. Consistent with earlier studies, this study could demonstrate a highly significant monocyte reduction, irrespective of the DT injection count. In line with other reports, different depletory effects on monocyte subsets were found, with Ly6C^low^ monocytes being the most affected, corroborating previous reports [53,54,55]. The mature Ly6C^low^ subset is assumed to derive from Ly6C^hi^ and consequently requires a longer regeneration time. When analyzing the FACS data, it is important to note that only relative frequencies, but not absolute numbers, were analyzed. Therefore, the apparent minor effect of DT on Ly6C^hi^ monocytes in CD11b-DTR mice is most likely due to the enormous stimulus elicited by the surgical procedure on the frequency of circulating Ly6C^hi^ monocytes. Similar to the distinct phenotypes of circulating monocytes, there is compelling evidence demonstrating that various MΦ subsets exist within the intestinal MΦ pool. These subsets are defined by surface markers or a distinct genetic profile and exist within a specific niche [8]. Among them are LP MΦ, mucosal perivascular macrophages, blood-vessel-associated MΦ or neuron-associated MΦ. Given the heterogeny of specialized macrophage subsets in the intestine, it is important to acknowledge that this study does not provide in-depth information regarding the susceptibility of these subsets towards DT-induced depletion, and it is possible that one subset is more susceptible to DT treatment than another, which could have impacted intestinal AH in our model. Accordingly, further research is needed to better define the subset-specific effect of DT treatment in CD11b-DTR mice.

The GSEA results indicate that MΦ function during intestinal AH is highly dynamic, evidenced by the vast changes in MΦ-associated pathways, ranging from orchestrating the inflammatory response and cytokine production as well as eliciting collagen catabolism (inflammatory phase) to regulating ECM assembly and facilitating collagen biosynthesis (reparative phase). The phase-specific and especially context-dependent roles of intestinal MΦ presented here are in line with recent data from Strowitzki et al., showing the positive effects of a decreased infiltration of M1-like MΦ and enhanced numbers of M2-line MΦ on AH under septic conditions [56]. Similar data have been presented by Neumann et al. in a model of inflammation-associated AH, suggesting the MΦ phenotypic switch from M1 to M2 to be a possible protective mechanism of action for annexin A1 [57]. The data presented here add to the existing concept of targeting an MΦ phenotype switch to improve intestinal AH by demonstrating that the MΦ function is dynamic, temporally defined, and evolves with the stages during AH. Additionally, the inflammatory and proliferative phases in particular represent a potential therapeutic window to enhance an M2-like phenotype in MΦ without compromising intestinal AH. When interpreting the results in comparison to the data from Strowitzki et al. and Neumann et. al., one must point out that both groups used models of AH under inflammatory, ischemic, or septic conditions. Therefore, the used model of physiological AH might underestimate MΦ-elicited effects and further research is needed to expand the model of phase-dependent MΦ depletion to AH under pathological conditions. This is further supported by recent data showing that unlike under normal conditions, MΦ depletion under chronic inflammatory conditions (diabetes-impaired wound-healing) improved dermal wound healing [58].

One of the limitations of the current study is the lack of evaluation of colonic motility, since intestinal dysmotility and postoperative ileus are common and clinically relevant complications of gastrointestinal surgery, especially after the creation of an anastomosis. This is of special interest as MΦ have been linked to gastrointestinal motility as well as inflammation and surgery-related intestinal dysmotility [59,60]. Neuron-associated MΦ are located in close proximity to the myenteric plexus in the muscularis externa [8]. Initially, depletion of these muscularis-externa-resident MΦ has been shown to prevent postoperative ileus [61]. However, a recent study by Farro et al. demonstrated that a genetic reduction in monocyte-derived MΦ (using the CCR2 knockout mouse) impairs the resolution of inflammation in the muscularis externa and thus increases neuronal damage and delays recovery from postoperative ileus [62]. Therefore, tissue-resident and recruited monocyte-derived MΦ can have opposite roles during the initiation and resolution of inflammation. This in line with results from this study, since it further demonstrates that targeting MΦ must always balance their dual and temporally defined role in inflammation and resolution as well as tissue repair.

This study also adds considerable evidence to the concept that as wounds heal, the local MΦ population transitions from predominantly pro-inflammatory (M1-like) to anti-inflammatory and pro-resolving (M2-like) phenotypes. From a translational perspective, this is of importance since chronic skin wounds are characterized by the prolonged persistence of pro-inflammatory MΦ and diminished wound healing has been associated with a delayed MΦ switch from M1 to M2 [63]. Impaired intestinal AH is especially frequent in IBD patients, which are at higher risk for AL but also suffer frequently from fibrosis, which also has been identified to be MΦ-dependent. One of the key drivers of intestinal fibrosis is *TGF-β*, which is secreted by M2 MΦ. The observed decrease in *TGF-β* in CD11b-DTR mice during the reparative phase of AH demonstrates M2-like MΦ to be significantly present during this phase, but also suggests that reducing or antagonizing *TGF-β* during the later stages of AH to counteract intestinal fibrosis is possible without compromising AH. However, recent data from Salvador et al. demonstrated that the M2a MΦ subset in particular reduced intestinal fibrosis elicited by chronic intestinal inflammation [64]. So far, there are insufficient data regarding the exact MΦ subset that elicit profibrotic or fibrolytic effects during intestinal AH. However, the available data suggest that alterations in the intestinal MΦ compartment, regardless of the phenotype, can lead to wound healing complications ranging from AL to fibrosis. Therefore, any therapeutic intervention (irrespective of aiming at preventing AL or intestinal fibrosis) must critically balance the distinct and dual roles that MΦ take on to ensure proper intestinal AH.

## 5. Conclusions

In conclusion, this study is the first to provide evidence for MΦ-associated pathways using a temporally restricted MΦ depletion strategy during distinct phases of intestinal AH. This study demonstrates that MΦ depletion has a profound impact on phase-specific repair mechanisms, without compromising AH. The data presented here suggest that MΦ reduction, selectively during early wound repair could attenuate the inflammatory response, without altering wound closure, which might be clinical of interest, especially in IBD.

## Data Availability

The data presented in this study are available on reasonable request from the corresponding author.

## Supplementary Materials

The following supporting information can be downloaded at: https://www.mdpi.com/article/10.3390/cells12071039/s1, Figure S1: Technique of murine colonic end-to-end anastomosis; Table S1: List of 422 genes related to wound healing (GO term 0042060 “wound healing”); Table S2: List of top 50 differently regulated genes related to wound healing (GO term 0042060 “wound healing”) per timepoint.
