# Supplementary material for: The Impact of Phase-Specific Macrophage Depletion on Intestinal Anastomotic Healing
Source: Cells. 2023 Mar 29;12(7):1039. doi: 10.3390/cells12071039 (PMC10093464; doi:10.3390/cells12071039)
Supplement: Supplementary file 1 [file cells-12-01039-s001.zip › cells-2279490-supplementary.pdf]

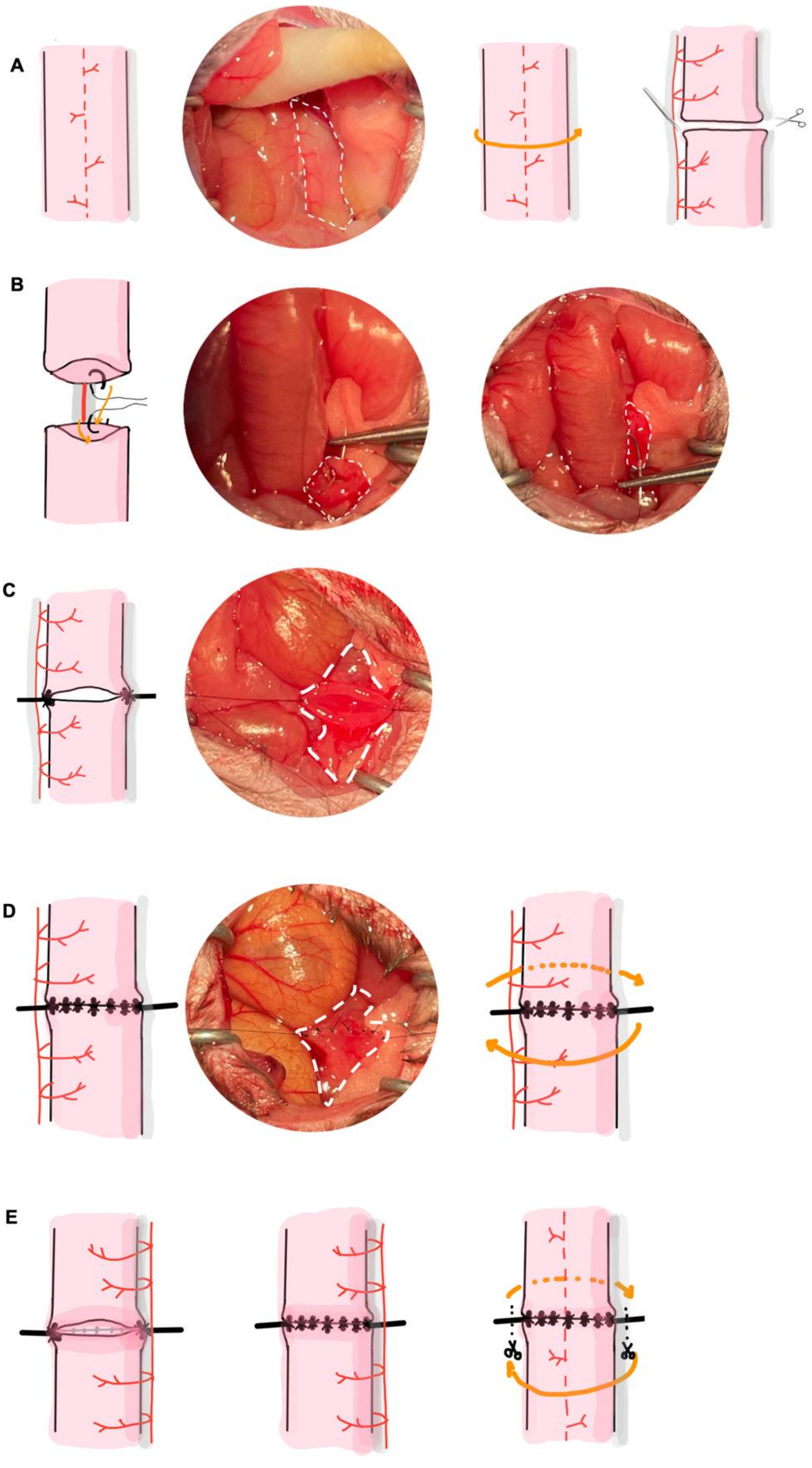

Supplementary Figure S1. Technique of murine colonic end-to-end anastomosis.
(A) Exposure of colonic rectosigmoid junction (**A1**, scheme; **A2**, photo, dashed line outlines colonic walls). **A3**, scheme, 90°rotation of colon to **A4,** scheme, secure colonic artery in the mesocolon during transection of the colon. **(B)** End-to-end anastomosis starts with one anchor stitch next to mesocolon and one anchor stitch opposite of mesocolon (**B1**, scheme, orange arrows indicate needle guidance; **B2-3**, photos). **(C)** Colonic lumen is stretched between anchor threads (**C1**, scheme; **C2**, photo, dashed line outlines colon). **(D)** 5 single button stitches on the anterior side (**D1**, scheme; **D2**, photo, dashed line outlines colon). **(E)** 180°rotation to **E1**, scheme, posterior side of the colonic lumen to **E2**, scheme, set 5 more single button stitches; followed by **E3**, scheme, removal of anchor threads and reposition of colon.

Supplementary Table S1.
List of 422 genes related to wound healing (GO term 0042060 “wound healing”) .

Abat, Abcc8, Acta2, Actb, Actg1, Actn1, Acvrl1, Adam17, Adamts13, Adamts18, Adipor2, Adora2a, Adra2a, Adra2b, Adra2c, Adtrp, Ajap1, Ajuba, Ak3, Alox12, Alox15, Alox5, Ano6, Anxa1, Anxa5, Anxa6, Anxa8, Ap3b1, Apcs, Apoe, Apoh, Arfgef1, Arhgap24, Arhgap35, Arhgef19, Arl8b, Axl, B4galt1, Blk, Bloc1s3, Bloc1s4, Bloc1s6, C1galt1c1, C1qtnf1, BC004004, Cadm4, Carmil2, Cask, Casp3, Cav1, Cav3, Ccn1, Ccn4, Cd109, Cd151, Cd34, Cd36, Cd40, Cd40lg, Cd44, Cd59b, Cd9, Cdh3, Cdkn1a, Ceacam1, Cela2a, Cflar, Chmp1a, Chmp1b, Chmp2a, Chmp2b, Chmp3, Chmp4b, Chmp4c, Chmp5, Chmp6, Chmp7, Clasp1, Clasp2, Cldn1, Cldn19, Cldn3, Cldn4, Clec7a, Clic1, Cnn2, Col1a1, Col3a1, Col5a1, Comp, Coro1b, Cpb2, Crk, Csrp1, Ctsg, Cx3cl1, Cxcr4, Cyp4f14, Cyp4f15, Cyp4f40, Cyp4f14, Cyp4f15, Dag1, Dcbld2, Ddr1, Dgka, Dgkb, Dgkd, Dgke, Dgkg, Dgkh, Dgki, Dgkk, Dgkq, Dgkz, Dmtn, Drd5, Dsp, Dst, Dtnbp1, Duox1, Duox2, Dysf, Edn1, Elk3, Emilin2, Enpp4, Entpd1, Entpd2, Epb41l4b, Ephb2, Eppk1, Erbb2, Erbb3, Ereg, Evpl, Ext1, F10, F11, F11r, F12, F13a1, F13b, F2, F2r, F2rl1, F2rl2, F2rl3, F3, F5, F7, F8, F9, Fap, Fbln1, Fcer1g, Fer1l5, Fermt1, Fermt2, Fermt3, Fga, Fgb, Fgf1, Fgf10, Fgf2, Fgfr1op2, Fgfr2, Fgg, Fibp, Fkbp10, Flna, Fn1, Foxa2, Foxc2, Fundc2, Fut10, Fzd6, Fzd7, Gas6, Gata1, Gata2, Gata4, Ggcx, Gli3, Gna12, Gna13, Gnaq, Gnas, Gp1ba, Gp1bb, Gp5, Gp6, Gp9, Gpr4, Gpx1, Grhl3, Hbb-bt, Hbegf, Hgfac, Hif1a, Hmox1, Hnf4a, Hps4, Hps5, Hps6, Hpse, Hras, Hrg, Hspb1, Igf1, Il1a, Il24, Il6, Il6st, Ilk, Ins2, Insl3, Itga2, Itga5, Itgb1, Itgb3, Itgb6, Itpk1, Jak2, Jaml, Jmjd1c, Kank1, Kdr, Klkb1, Kng2, Krt1, Krt6a, Lck, Lcp1, Lman1, Lnpk, Lox, Lyn, Macf1, Map3k5, Mapk14, Mcam, Mertk, Metap1, Mia3, Mmp12, Mmrn1, Mpig6b, Mpl, Mrtfa, Msx2, Mtor, Myh9, Myl12a, Myl9, Mylk, Myof, Myoz1, Ndnf, Nf1, Nfe2l2, Nlrp6, Nog, Nos3, Notch2, Notch4, Nrg1, Ocln, Odam, Oprm1, P2rx1, P2ry1, P2ry12, Pabpc4, Pafah2, Pak1, Papss2, Pdcd10, Pdgfa, Pdgfb, Pdgfra, Pdgfrb, Pdpn, Pear1, Pf4, Pf4, Phldb2, Pik3ca, Pik3cb, Pik3cg, Pla2g4a, Plat, Plau, Plaur, Plcg2, Plec, Plek, Plet1, Plg, Plpp3, Plscr1, Ppara, Ppard, Pparg, Ppia, Ppl, Ppp3ca, Prcp, Prdx2, Prkca, Prkcd, Prkce, Prkcq, Prkg1, Proc, Procr, Pros1, Proz, Prss56, Pten, Ptk2, Ptk7, Ptpn6, Rab27a, Rab3a, Raf1, Rap2b, Reg3b, Reg3g, Rhoa, Rhoc, Rreb1, S100a8, Saa1, Saa2, Scarb1, Scrib, Scube1, Sdc1, Sdc4, Selp, Serpina1b, Serpina1e, Serpina10, Serpinb2, Serpinc1, Serpind1, Serpine1, Serpine2, Serpinf2, Serping1, Sh2b3, Shh, Slc11a1, Slc12a2, Slc4a1, Slc7a11, Smad2, Smad3, Smad4, Smoc2, Smpd1, Sprr3, Src, Srf, Srsf6, St3gal4, Stxbp1, Stxbp3, Syk, Syt11, Syt7, Sytl4, Tafa5, Tbxa2r, Tec, Tfpi, Tfpi2, Tgfa, Tgfb1, Tgfb2, Tgfb3, Tgfbr1, Tgfbr2, Thbd, Thbs1, Timp1, Tln1, Tlr4, Tmeff2, Tnf, Tnfaip3, Tnfrsf12a, Tor1a, Tpm1, Treml1, Trim72, Tsku, Tspan32, Tspan8, Txk, Tyro3, Ubash3b, Usf1, Vav1, Vav2, Vav3, Vcl, Vegfa, Vil1, Vkorc1, Vtn, Vwf, Was, Wfdc1, Wnt3a, Wnt4, Wnt5a, Wnt5b, Wnt7a, Xbp1, Yap1

Supplementary Table S2.
List of top 50 differently regulated genes related to wound healing (GO term 0042060 "wound healing") per timepoint.

Inflammatory phase: Metap1, Stxbp3, Pafah2, Vegfa, Fibp, Ap3b1, Vcl, Reeb1, Pdgfa, Map3k5, Adam17, Cd44, Scarb1, P2ry1, Plat, Fermt3, Lox, Syt11, Plet1, Igf1, Cxcr4, St3gal4, Crk, Arfgef1, B4galt1, Apoe, Cldn4, Rhoc, Cdkn1a, Alox5, Mpig6b, Lck, Cd40, Cdh3, Carmil2, Saa2, Tspan32, Blk, Cadm4, Ubash3b, Tnf, Clec71, Ndnf, Grhl3, F5, P2ry12, P2rx1, Alox15, Comp, Gp5

Proliferative phase: Dgkd, Arhgap35, Dst, Syk, Syk7, Duox2, Rreb1, Prkca, Ephb2, Pdgfa, Hras, Chmp2a, Cd151, Chmp5, Mia3, Arfgef1, Prdx2, Chmp2b, Chmp1a, Fibp, BC004004, Usf1, Chmp7, Cflar, Serpine2, Tor1a, St3gal4, Cldn3, Tspan8, Cd9, Myh9, Gpx1, Chmp4b, F11r, Saa1, Adra2a, Vkorc1, Bloc1s4, Vav2, Entpd2, Plcg2, Pik3cg, Mmp12, Slc7a11, Ubash3b, Abcc8, F7, Cd59b, Cldn1, Saa2

Reparative phase: Map3k5, Prcp, Srf, Adam7, Itga5, Gnaq, Ephb2, Chmp6, Fibp, Usf1, Tor1a, Clasp1, St3gal4, Notch2, Clasp2, Itga2, Hbegf, Wfdc1, Ggcx, Sytl4, Plcg2, Arhgap24, Ccn1, Rab3a, Vav2, Serpine2, Dgke, Jaml, Ndnf, Ubash3b, Txk, Mia3, Tln1, Actn1, Hgfac, Fermt1, Chmp2a, Arhgap35, Vcl, Macf1, Duox2, Cyp4f14, Anxa8, Chmp1a, Dag1, Chmp4b, Flna, Col3a1, Tpm1, Saa1
